# Validation of Qualitative Broth Volatilization Checkerboard Method for Testing of Essential Oils: Dual-Column GC–FID/MS Analysis and In Vitro Combinatory Antimicrobial Effect of *Origanum vulgare* and *Thymus vulgaris* against *Staphylococcus aureus* in Liquid and Vapor Phases

**DOI:** 10.3390/plants10020393

**Published:** 2021-02-18

**Authors:** Marie Netopilova, Marketa Houdkova, Klara Urbanova, Johana Rondevaldova, Ladislav Kokoska

**Affiliations:** 1 Department of Crop Sciences and Agroforestry, Faculty of Tropical AgriSciences, Czech University of Life Sciences Prague, 16500 Praha, Czech Republic; netopilova@ftz.czu.cz (M.N.); houdkovam@ftz.czu.cz (M.H.); rondevaldova@ftz.czu.cz (J.R.); 2 Department of Sustainable Technologies, Faculty of Tropical AgriSciences, Czech University of Life Sciences Prague, 16500 Praha, Czech Republic; urbanovak@ftz.czu.cz

**Keywords:** antimicrobial interactions, broth volatilization chequerboard method, chemical composition, gaseous phase, fractional inhibitory concentration, GC/MS, oregano, thyme, volatile compound

## Abstract

Combinatory action of antimicrobial agents such as essential oils (EOs) show to be an effective strategy to overcome the problem with increasing antibiotic resistance of microorganisms, including *Staphylococcus aureus*. The objective of this study was to evaluate in vitro antimicrobial interactions between *Origanum vulgare* and *Thymus vulgaris* EOs against various *S.* *aureus* strains in both liquid and vapor phases using the broth volatilization checkerboard method. Fractional inhibitory concentrations (FICs) were determined for both liquid and vapor phases, and the composition of EOs was analyzed by gas chromatography-mass spectrometry using dual-column/dual-detector gas chromatograph. Results of oregano and thyme EOs combination showed additive effects against all *S. aureus* strains in both phases. In several cases, sums of FICs were lower than 0.6, which can be considered a strong additive interaction. The lowest FICs obtained were 0.53 in the liquid phase and 0.59 in the gaseous phase. Chemical analysis showed that both EOs were composed of many compounds, including carvacrol, thymol, *γ*-terpinene, and *p*-cymene. This is the first report on oregano and thyme EOs interactions against *S. aureus* in the vapor phase. It also confirms the accuracy of the broth volatilization checkerboard method for the evaluation of combinatory antimicrobial effects of EOs in the vapor phase.

## 1. Introduction

*Staphylococcus aureus* is a gram-positive bacterium that has been responsible for a broad spectrum of diseases, ranging from food poisoning and superficial skin and soft tissue infections to life-threatening infections such as bacteremia, endocarditis, osteomyelitis, pneumonia, or toxic shock syndrome [1]. It is notorious for its ability to quickly become resistant to any antibiotic, which makes this bacterium one of the most serious pathogens in humans, and its treatment is often difficult [2]. In humans, *S. aureus* can occur as both a benign commensal and a harmful pathogen. Besides being a common colonizer of the skin, it also asymptomatically and permanently colonizes the anterior nostrils of up to 30% of the normal human population [3,4], which is widely considered to be a predisposition of invasive infection [5]. Since *S. aureus* is a microorganism that is associated with a broad spectrum of infections affecting the respiratory tract, taking up antibiotics through inhalation could be one of its possible treatments. Moreover, the combination of two or even more antibiotic agents may be used as a possible strategy for increasing efficiency in fighting *S. aureus*-related diseases, including respiratory infections. An example of such treatment is a combination of fosfomycin and tobramycin that is currently in the late-stage development of an inhalation therapy of cystic fibrosis [6,7]. It was demonstrated that fosfomycin synergistically enhances the activity of tobramycin against a wide range of bacteria, including *S. aureus* [6,8]. However, inhalation of solid antimicrobial agents, as well as the use of inhaler devices, may often be problematic, especially in children and the elderly [9]. Therefore, there is a need to search for new antimicrobial agents to combat bacteria affecting the respiratory tract and for easier ways how to deliver antimicrobials into the lower respiratory tract.

Recently, as concerns about the increasing bacterial resistance to conventional antibiotics are growing, the use of medicinal plants, their unique properties, and possibilities of applications are more frequently proposed as an option for treating these problems. The use of agents of complex chemical composition, such as essential oils (EOs), as well as the therapy combination based on a combination of drugs, have already shown to be generally effective strategies to overcome issues with microbial resistance. In general, the antibacterial activity of any EO may depend on one major compound only; however, new findings show that interactions with other compounds in the oils are also important [10], whereas possible synergistic or antagonistic effects between EO constituents can either enhance or reduce activities of single compounds [11]. Similarly, EOs, when used in combination, can initiate a synergistic antimicrobial effect. Various experiments focused on interactions between EOs and their volatile constituents have previously been conducted against numerous microorganisms. In the extensive review of Leigh-de Rapper and van Vuuren [12] that was focused on EOs against pathogens of the respiratory tract, synergy was determined for 34% of the EOs combinations. However, only a limited number of studies dealing with the combinatory effects have been performed in the vapor phase, as recently reviewed in Houdkova and Kokoska [13]. One of the reasons is that there is only a limited number of assays suitable for qualitative evaluation of antimicrobial interactions of volatile agents in the gaseous phase. Recently, we have developed a new broth volatilization checkerboard method allowing evaluation of the antimicrobial activity of volatile agents in the vapor phase, of which the accuracy was verified on a combination of two plant-derived compounds [14] as well as on the combination of a compound with an EO [15]. However, the usability of this method for the determination of the combinatory antimicrobial effect of two EOs has not been proven yet.

*Origanum vulgare* L. (oregano) and *Thymus vulgaris* L. (thyme) are aromatic spice herbs belonging to the Lamiaceae family. They are native to the Mediterranean region and neighboring countries [16,17], and in folk medicine, they have been used as remedies to treat respiratory disorders (e.g., coughs and bronchitis) as expectorants, dyspepsia as well as urinary tracts disorders [16,17,18,19]. Although the principal components of oregano and thyme EOs are carvacrol and thymol, respectively, their chemical compositions vary depending on geographical region and season of collection [20]. Both plants are also used in pharmaceutical industries, including the products for the treatment of respiratory infections. For example, the extract of *T. vulgaris* is used in two oral over-the-counter products Bronchipret Saft and Bronchipret TP (Bionorica, Neumarkt, Germany), which are used for the treatment of respiratory tract illnesses, cough, and bronchitis [21]. Furthermore, EOs derived from these plants have been shown to exhibit a broad range of considerable biological activities, including their antimicrobial effect, which has been mostly attributable to the presence of phenolic compounds, such as carvacrol and thymol [22,23,24]; however, other minor constituents such as monoterpene hydrocarbons *γ*-terpinene and *p*-cymene contribute to the antibacterial activity of the oils as well [22,24]. To date, numerous studies concerned with the chemical composition and antimicrobial activity of *O. vulgare* and *T. vulgaris* EOs have been published. Differences between the antimicrobial activities of various chemotypes of these oils have been described [25,26]. Furthermore, it was also proved that the antimicrobial effect of these EOs might be comparable to their main component alone [27]. Due to their antimicrobial properties, EOs (including *O. vulgare* and *T. vulgaris* EOs) could be used as alternatives to conventional antimicrobial agents, especially against antibiotic-resistant pathogens [24]. So far, numerous studies regarding the antibacterial activity of *O. vulgare* and *T. vulgaris* EOs alone against a wide range of microorganisms, including *S. aureus,* have been published [28,29,30]. Both EOs have also previously been tested against *S. aureus* in combination with other EOs [31,32] as well as with classic/conventional antibiotics [33]. Moreover, their synergistic and additive inhibitory activity with each other has previously been reported against *S. aureus* as well [34,35]. However, although there are numerous articles on the antistaphylococcal activity of *O. vulgare* and *T. vulgaris* EOs tested in the broth and agar, substantially fewer articles dealing with their antibacterial effects against *S. aureus* have been published using the vapor phase [36,37]. Moreover, to the best of our knowledge, the combinatory antistaphylococcal activity of *O. vulgare* EO and *T. vulgaris* EO have not previously been studied in the gaseous phase.

Based on the results of our preliminary screenings performed as several combinations of different EOs (*Cinnamomum cassia*, *C. verum*, *Cymbopogon flexuosus*, *O. vulgare*, *Syzygium aromaticum*, and *T. vulgaris*) against *S. aureus* of the American Type Culture Collection (ATCC) 29213 (the lowest fractional inhibitory concentration (FIC) values in the vapor phase ranged from 0.59 to 1.25), the combination of *O. vulgare* EO with *T. vulgaris* EO was selected for more detailed evaluation due to its lowest FIC values that it had produced (unpublished data). Therefore, the aim of the present study was to determine the antibacterial combinatory potential of EOs hydrodistilled from *O. vulgare* and *T. vulgaris* against standard strains and clinical isolates of *S. aureus* in both the vapor and liquid phases. Since the methods currently available for the determination of antimicrobial interactions of EOs in the vapor phase are based on disk diffusion assay, which yields only qualitative information about the antimicrobial agent combination, the accuracy of these techniques is limited because it is difficult to distinguish indifferent from synergistic interaction. For this reason, the validation of the qualitative broth volatilization checkerboard method for testing of combinatory antimicrobial effect of two different EOs was an additional objective of this study.

## 2. Results

### 2.1. Antimicrobial Analysis

The detailed results of individual minimum inhibitory concentrations (MICs) of *O. vulgare* and *T. vulgaris* EOs against 12 strains of *S. aureus* including clinical isolates, as well as the MICs of their combinations with corresponding ΣFIC values are summarized in Table 1 and Table 2 for the vapor and liquid phases, respectively. Results show that *O. vulgare* EO exhibited an antistaphylococcal effect with MICs ranging from 427 to 796 μg/mL and from 512 to 1024 μg/mL in agar and broth media, respectively. Similar numbers were observed for *T. vulgaris* EO with MICs ranging from 427 to 796 μg/mL in the vapor phase and from 512 to 967 μg/mL in the liquid phase.

**Figure 1:**
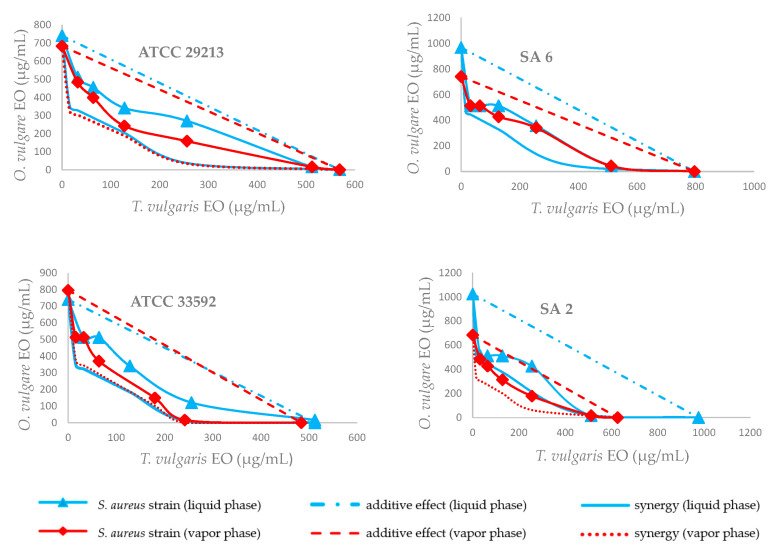
Isobolograms of the interactions between *Origanum vulgare* and *Thymus vulgaris* binary combinations against *Staphylococcus aureus* strains in vapor and liquid phases. Additivity (ΣFIC > 0.5 and ≤ 1); synergy (ΣFIC ≤ 0.5).

Considering their combinatory activity, EO of *O. vulgare* in combination with *T. vulgaris* EO produced an additive antimicrobial effect against all 12 strains tested. The combination profiles of four *S. aureus* strains are presented graphically in Figure 1. The isobole curves clearly show the additive effect against *S. aureus* strains tested, whereas the additive interactions can be read according to the curves indicating the borderline of additivity and synergy. In several cases (i.e., for one combination of these volatile agents in the vapor phase and four combinations in broth), they showed ΣFICs lower than 0.6, which can be considered a strong additive interaction, reaching values close to the synergistic effect. The most effective concentrations inhibiting the growth of *S. aureus* (SA) were found in the liquid phase against methicillin-resistant clinical isolate SA 2 at 512 μg/mL of *O. vulgare* EO and 32 μg/mL of *T. vulgaris* (ΣFIC = 0.53) and in the vapor phase against standard strain SA ATCC 29213 at 242 μg/mL of *O. vulgare* EO and 128 μg/mL of *T. vulgaris* EO (ΣFIC = 0.59). On average, the best FIC values were observed in both the liquid and vapor phases when the concentrations of *T. vulgaris* EO were 256 and 128 μg/mL. Based on the results, the optimum ratio of *T. vulgaris* and *O. vulgare* to achieve bacterial inhibition would be 0.5-2:1 in the vapor phase and 0.4-1.2:1 in the liquid phase.

### 2.2. Gas Chromatography/Mass Spectrometry (GC/MS) Analysis

The yields of *O. vulgare* and *T. vulgaris* EOs in the dried weight of plant materials (containing 14.42% and 13.68% of residual moisture) for *T. vulgaris* were 1.5% and 1.2% (*v/w*), respectively. The complete chemical compositions of oregano and thyme EOs are provided in Table 3; Table 4, respectively. In EOs isolated from *O. vulgare* and *T. vulgaris*, 19 and 28 components have been identified using an HP-5MS column, representing 99.78% and 99.26% of their respective total content. Using DB-HeawyWAX column, 25 and 34 compounds were determined, which constitute 99.90% and 99.53% of the volatile oil, respectively. In total, 26 compounds were identified in the EO of *O. vulgare*, whereas 37 compounds were found in the EO isolated from *T. vulgaris*. The analysis showed that the most monoterpene hydrocarbons and oxygenated monoterpenes were the main groups of chemicals in both EOs.

In EO extracted from *O. vulgare*, carvacrol was the predominant compound representing 77.92%/82.60% (= 13.52/15.01 mg/kg) when measured using HP-5MS/DB-HeawyWAX columns, respectively. Other compounds detected in significantly lower amounts were *p*-cymene and *γ*-terpinene with percentage values 8.25%/5.63% (= 1.24/0.88 mg/kg) and 4.52%/3.33% (= 0.74/0.57 mg/kg). In EO obtained from *T. vulgaris*, thymol was the most abundant component representing 42.34%/48.46% (= 6.55/10.04 mg/kg), followed by *p*-cymene and *γ*-terpinene representing 24.08%/18.00% (= 3.22/3.22 mg/kg) and 13.37%/10.61% (= 1.96/2.08 mg/kg) when measured using HP-5MS/DB-HeawyWAX columns, respectively.

## 3. Discussion

In our study, the in vitro growth inhibitory effect of both *O. vulgare* and *T. vulgaris* EOs was slightly stronger in the vapor phase than in a liquid medium since the MIC values were for the vast majority of the staphylococcal strains slightly lower on the agar media than in the broth. The only exceptions were standard strain ATCC 33592 and clinical isolate SA 1, where the antimicrobial effect of *O. vulgare* was stronger in the liquid phase, and staphylococcal strains ATCC 29213, ATCC 43000, SA 1, and SA 6, where the MICs of *T. vulgaris* EO were the same in both phases. Similar pattern showing that the vapor generated by EOs has a greater antimicrobial effect compared to EOs in liquid form applied by direct contact (in aqueous solutions or on solid agars) can be observed in several previous studies [48,49]. This phenomenon can be explained by the fact that in the aqueous phase, lipophilic molecules associate to form micelles and thus restrain the attachment of EOs to microorganisms, whereas the vapor phase allows for free attachment [50,51].

Values of MICs observed in our study for *O. vulgare* and *T. vulgaris* EOs in the liquid phase were similar to numerous previously published data. For example, the investigation carried out by Boskovic et al. [30] determined antibacterial effects of EOs isolated from *O. vulgare* against *S. aureus* ATCC 25923 (MIC values = 640 μg/mL) and methicillin-resistant *S. aureus* (MRSA) ATCC 43300 (MIC values = 320 μg/mL) using broth microdilution method. In the study performed by Ozkalp et al. [28], *O. vulgare* EO inhibited growth of *S. aureus* Refik Saydam National Type Culture Collection 96090 and MRSA with MIC values 64 and 250 μg/mL, respectively. Similarly, the results of antistaphylococcal activity of *T. vulgaris* EO obtained in this study correspond well with previous findings of Kot et al. [52], who reported MIC values ranging from 90 to 780 μg/mL against 18 MRSA strains in the liquid phase, or with the results of Boskovic et al. [30], who determined the antimicrobial effect of thyme EO against *S. aureus* ATCC 25923 (MIC values = 640 μg/mL) and MRSA ATCC 43300 (MIC values = 320 μg/mL). Moreover, our results are supported by research conducted by Stojkovic [34], where MICs of oregano and thyme EOs against *S. aureus* were equal to 0.5 μL/mL and 1 μL/mL, respectively.

If mixtures of EOs are used as antimicrobials, they may, according to the European Committee on Antimicrobial Susceptibility Testing (EUCAST) [53], show either an antagonistic, additive, indifferent, or synergistic effect, measured by assessment of the FIC values. Several authors have demonstrated additive effects as well as synergistic actions of *O. vulgare* EO in combination with *T. vulgaris* EO in the liquid phase. Our results correspond well with Gavaric et al. [35], who reported additive antibacterial action of thyme and oregano against several bacteria, including *S. aureus* ATCC 25923 (FIC value = 1). Similarly, Gutierrez et al. [54] confirmed the additive effect of these EO combinations against several spoilage organisms, such as *Enterobacter cloacae*, *Pseudomonas fluorescens,* and *Listeria innocua,* using a checkerboard method with FIC values ranging from 0.75 to 1. On the other hand, synergistic activity of oregano and thyme EO combinations have previously been reported as well; for example, in the study of Stojkovic et al. [34], oregano combined with thyme EO produced synergy against *S. aureus* (FIC value 0.45). However, as the assayed EOs possess similar chemical composition, their combination may exhibit addition rather than a synergistic effect [55].

The disc volatilization method is probably the most frequently used assay for the evaluation of in vitro growth inhibitory effect in the vapor phase. Both EOs have previously been tested individually against *S. aureus* in the gaseous phase. For example, an investigation carried out by Nedorostova et al. [37] determined antibacterial effects of EOs isolated from *O. vulgare* and *T. vulgaris* against *S. aureus* ATCC 25923 using modified disc volatilization method, and the MIC value of both EOs was 0.017 μg/cm^3^. Similarly, Kloucek et al. [56] used a modified disc volatilization method to assess the antimicrobial activity of various EOs, including those of oregano and thyme. In this study, vapors of *O. vulgare* EO containing 92% of carvacrol inhibited growth of *S. aureus* ATCC 25923 with MIC values 62.5 μL/L, whereas three EO samples of *T. vulgaris* with different chemical composition exhibited antimicrobial activity against the same staphylococcal strain with the MIC ranging from 125 to 250 μL/L. However, a thyme EO where thymol was the predominant compound was not active at all. A study performed by Lopez et al. (2007) [36] determined the growth inhibitory effects of *O. vulgare* and *T. vulgaris* vapors against *S. aureus* ATCC 29213 by a similar method and consequently calculated MIC causing apparent inhibition (17.5 μL/L and 87.3 μL/L, respectively) of the atmosphere above microorganisms. Subsequent research led by Reyes-Jurado et al. [57] assessed the MIC values of *T. vulgaris* EO vapors against *S. aureus* and MRSA as >5 μg/mL of air. However, although there has been increasing research interest in the antimicrobial activity of individual EO vapors in recent years, significantly fewer studies have been reported on their combinations. In the case of thyme and oregano EO vapors, to the best of our knowledge, the only study dealing with their combinatory effects in the gaseous phase was published by Cho et al. [58], who reported synergistic activities of gaseous oregano and thyme EOs against *Listeria monocytogenes* by modified checkerboard assay (FIC = 0.375). Our study is the first report on *O. vulgare* and *T. vulgaris* interactions in the vapor phase against *S. aureus*.

The antimicrobial properties of *O. vulgare* and *T. vulgaris* have been attributed to their chemical compositions, which are primarily rich with monoterpene hydrocarbons and oxygenated monoterpenes. The principal terpenes identified in oregano and thyme are usually carvacrol, thymol, *γ*-terpinene, and *p*-cymene; while terpinen-4-ol, linalool, *β*-myrcene, *trans*-sabinene hydrate, and *β*-caryophyllene are also present. The proportion of these and other components in oils within the same species defines the chemotype [59]. In our study, the chromatographic profiles of both EOs were analyzed by GC/MS using two detectors and two capillary columns of different polarities to avoid the overlapping of signal peaks observed in the chromatogram and to achieve the best possible resolution of compounds. The internal standard was used for quantitative analysis. Compounds belonging to the classes with monoterpene hydrocarbons and oxygenated monoterpenes were the most numerous identified. Carvacrol was the most abundant compound in oregano EO, followed by *p*-cymene and *γ*-terpinene, and the oil is, thus, characterized as a carvacrol chemotype. This finding is in accordance with several previously published studies. For example, Stojkovic et al. [34], Scalas et al. [60], and Stoilova et al. [61] reported carvacrol as the main component of oregano EO (contributing 64.50%, 62.61%, and 66.20% of the EO, respectively), *p*-cymene as the second the most abundant compound (10.90%; 12.36%, and 9.1%, respectively), and *γ*-terpinene as third most abundant component (10.80%, 7.60%, and 7.30%, respectively). Thymol, on the other hand, has been in our study detected as the most abundant constituent in thyme EO, also followed by its precursors, *p*-cymene and *γ*-terpinene; therefore the present thyme oil belongs to thymol chemotype. This finding is also in accordance with numerous previously published studies [26,29,30,34,62,63], where thymol, *p*-cymene, and *γ*-terpinene were reported as the first, second, and third most abundant compounds, respectively. The number of components identified in our study (26 and 37 in total in oregano and thyme EOs, respectively) is within the range of numbers of compounds identified in other reported studies, as eight to 38 compounds have been reported for *O. vulgare* [30,34,60,61,64], and 16–50 for *T. vulgaris* [26,29,30,34,60,62,63,65]. Since the used plant material has been obtained from a commercial supplier, the age of the plants as well as their growing conditions, harvest time, transportation, and storage conditions are unknown. Therefore, the chemical composition of EOs analyzed in this study can be influenced by all the above-mentioned factors [66,67]. The qualitative differences (numbers of components) between the two columns are in accordance with previously reported studies on GC/MS analysis of EOs using two columns. For example, Anderson and Parnell [68], who compared cold-pressed orange oil profiles by GC/MS using polar (Zebron ZB-WAX column) and non-polar (Zebron ZB-1ms) GC columns, identified 22 and 29 components on non-polar and polar compounds, respectively. The higher number of volatile components identified on a polar column might have been caused, similarly as in our case, by the better resolution between compounds that were seen to co-elute on the non-polar column. Similarly, quantitative differences between the polar and non-polar columns have previously been reported as well. In our study, the main compound (thymol) in thyme EO showed the highest proportional difference between two columns (more than 6%), which can be, for example, compared to Fan et al. [69], who analyzed the composition of the EO from *Dendranthema indicum* var. *aromaticum* and detected *α*-thujone as the main compound with a difference of 4.88% between columns. Different amounts of the detected compounds are caused by different polarity and material of the used columns. Besides determining of raw percentages of peak areas, the concentration of components in 1 kg of dry plant material was calculated using predicted relative response factors with an objective to increase the reliability and accuracy of the volatile components’ quantification [70]. This approach is important in technological processes with various applications in the field of chemical analysis of volatile plant-derived products because it allows the quantification of volatile components by GC/MS with flame-ionization detection when the authentic components are not available, and in addition, it can avoid time-consuming procedures of calibration [71].

Since carvacrol and thymol have been found to be the most abundant compounds in our oregano and thyme EOs, respectively, the additive effects obtained by interactions between our volatile oils might be caused mainly by these two phenolic monoterpenoids. The presumption that the predominant component in both EOs is responsible for the antimicrobial activity of EOs can be supported by our previous research [14], whereas the range of MIC values of carvacrol (370–1593 μg/mL and 484–1024 μg/mL in agar and broth media) and thymol (341–1707 μg/mL and 355–1024 μg/mL in the vapor and liquid phases) were very similar to the MIC values of the *O. vulgare* (427–796 μg/mL and 512–1024 μg/mL in agar and broth media) and *T. vulgaris* (427–796 μg/mL in the vapor phase and from 512–967 μg/mL in liquid phase) EOs tested in this study. The occurrence of additive interaction between carvacrol and thymol could be related to the similarity in their molecular structures (they are isomers), suggesting a similar mechanism of action [72]. Both thymol and carvacrol are expected to cause functional and structural damages to the cytoplasmic membranes. The primary mechanism of antibacterial action of thymol is not fully known; however, it is believed to involve outer and inner membrane disruption and interaction with membrane proteins and intracellular targets. Similarly, the primary mechanism of action of carvacrol is its ability to position in the membrane where it increases permeability [73]. In both EOs tested in our study, the principal compounds, carvacrol, and thymol, were followed by their biosynthetic precursors *p*-cymene and *γ*-terpinene, which, together with the main compound comprised more than 90% and 77% of the oregano and thyme oils. Their interaction within the tested EOs is presumable and might also contribute to the additive effects. This statement can be supported by Ultee et al. [74], who reported synergistic activity between carvacrol and cymene against *Bacillus cereus*, or by Delgado et al. [75], who found synergistic effect against the same bacterium when cymene was combined with thymol. The additive antimicrobial effect of carvacrol and thymol has already been previously reported in several studies against different bacteria, including *S. aureus* in liquid [35,76,77] as well as in the vapor phase [14]. However, further research focused on a better understanding of antimicrobial interactions between major and minor components, which was suggested to play an important role in the synergistic activity of EO gases [78] is warranted.

Although EO*s* of *O. vulgare* and *T. vulgaris* have acquired Generally Recognized as Safe (status from the Flavour and Extract Manufacturers Association and got approved by the US Food and Drug Administration (FDA) for safety food use [79,80]), there is limited published research on the safety of EO vapors *per se* [51]. As EOs are complex blends of components, individual volatile compounds need to be assessed as potential allergens. Currently, 26 ingredients that may trigger allergic reactions, including, e.g., linalool and limonene, are listed in the seventh amendment of directive 76/768 CEE (directive 2003/15/CE); however, these are all based on skin contact and not inhalation [81,82]. Regarding inhalation toxicity, which is a crucial aspect of inhalation administration, median lethal concentration (LC_50_) values were determined neither for oregano nor for thyme EOs for the inhalation route. However, the data on their predominant compounds, carvacrol and thymol, might suggest their possible inhalation safety. The European Chemicals Agency reported that the LC_50_ of carvacrol in rats was estimated to be greater than 20 mg/L when rats were treated with the given test chemical via inhalation route for 6 h exposure period. Similarly, the reported LC_50_ value for thymol was 7.57 mg/L, when mice were exposed to a test chemical via inhalation by vapor for 2 h [83]. Furthermore, neither data from literature nor results from chronic toxicity studies presented in the study by Xie et al. (2019) [84] provide any evidence for chronic toxicity of inhaled thymol. In an acute oral toxicity study, the median lethal dose (LD_50_) of carvacrol and thymol in rats was found to be 810 and 980 mg/kg of body weight (bw), respectively, and carvacrol-rich EO obtained from the leaves of *Origanum* spp. showed the oral LD_50_ to be 1850 mg/kg bw; therefore, they are all classified as category 4 (H302) according to the Classification, Labelling and Packaging Regulation N° 1272/2008 and the Globally Harmonized System of Classification and Labelling of Chemicals [83] which means that it might be “harmful if swallowed”. Moreover, thymol is FDA approved when used as a synthetic flavoring (21 CFR 172.515), a preservative and indirect food additive of adhesives [84], and is a common ingredient in many products such as perfumes, food flavorings, mouthwashes, pharmaceutical preparations, and cosmetics [85]. Similarly, carvacrol is generally considered safe for human consumption. It has been approved by FDA for its use in food and is included by the Council of Europe in the list of chemical flavorings that can be found in several food products, such as alcoholic beverages, baked goods, chewing gums, condiment relish, frozen dairy, gelatine puddings, non-alcoholic beverages, and soft candies [86]. Moreover, EO derived from *T. vulgaris* has been approved by the Committee on Herbal Medicinal Products of the European Medicines Agency as a traditional herbal medicinal product used for relief of cough associated with cold [87].

The above-mentioned data suggest a low toxicological risk of carvacrol and thymol administration through an inhalation route. Moreover, the rich historical evidence of culinary, medicinal, and pharmaceutical uses of *O. vulgare* and *T. vulgaris* could support their use as safe herbal medicinal products. Therefore, due to the considerable antimicrobial activity as well as the presumable safety of *O. vulgare* and *T. vulgaris* EOs, it can be assumed that the results of oregano and thyme EO combinations could be potentially applied in the development of various pharmaceutical applications that are based on volatile antimicrobials. These combinations could decrease the minimum effective doses of the agents, thus reducing their possible adverse effects and treatment costs. However, further research to achieve a better understanding of the action mechanisms, further in vivo experiments, and clinical trials on *O. vulgare* in combination with *T. vulgaris* are still necessary to determine their pharmacodynamics and pharmacokinetics.

## 4. Materials and Methods

### 4.1. Chemicals

Oxacillin (86.3%, Chemical Abstracts Service (CAS) number: 7240-38-2) and thiazolyl blue tetrazolium bromide (MTT, 98%, CAS: 298-93-1) were purchased from Sigma-Aldrich (Prague, Czech Republic), whereas dimethylsulfoxide (DMSO, CAS: 67-68-5) and *n*-hexane (CAS: 110-54-3) were obtained from Penta (Prague, Czech Republic). Methyl octanoate (≥99.8%, CAS: 111-11-5) and other standards (3-carene (99%, CAS: 498-15-7), borneol (97%, CAS: 464-45-9), bornyl acetate (95%, CAS: 5655-61-8), camphene (97.5%, CAS: 79-92-5), camphor (98%, CAS: 464-49-3), carvacrol (97%, CAS: 499-75-2), caryophyllene oxide (99%, CAS: 1139-30-6), linalool (97%, CAS: 78-70-6), *p*-cymene (99%, CAS: 99-87-6), thymol (99%, CAS: 89-83-8), *α*-pinene (≥99%, CAS: 7785-70-8), *α*-terpinene (85%, CAS: 99-86-5), *β*-caryophyllene (98.5%, CAS: 87-44-5), *β*-pinene (≥99.0%, CAS: 18172-67-3), and *γ*-terpinene (97%, CAS: 99-85-4)) were purchased from Sigma-Aldrich, Prague, Czech Republic.

### 4.2. Plant Material and Preparation of Essential Oils

The dried aerial parts of *O. vulgare* and *T. vulgaris* were purchased from a commercial supplier (U Salvatora, Prague, Czech Republic). Initially, they were homogenized by a Grindomix apparatus (GM100 Retsch, Haan, Germany). Subsequently, the residual moisture contents of both samples were determined gravimetrically at 130 °C for 1 h by Scaltec SMO 01 analyzer (Scaltec Instruments, Gottingen, Germany) in triplicate, and results were expressed as arithmetic averages according to the Official Methods of Analysis of the Association of Official Agricultural Chemists [88]. Both EOs were obtained by hydrodistillation of dried plant material in 1 L of distilled water using a Clevenger-type apparatus (Merci, Brno, Czech Republic) according to the procedure described in the European Pharmacopeia (2013) [89] and stored in sealed glass vials at 4 °C.

### 4.3. Bacterial Strains and Culture Media

In this study, 12 strains of *S. aureus* were used, including antibiotic-resistant and sensitive forms. Standard strains of the ATCC 25923, 29213, 33591, 33592, 43300, and BAA 976 were purchased from Oxoid (Basingstoke, UK) on ready-to-use bacteriological Culti-Loops. Clinical isolates (SA 1-6) obtained from Motol University Hospital (Prague, Czech Republic) were selected based on the previous antimicrobial susceptibility testing (data not shown) as representatives of methicillin-sensitive *S. aureus* (SA 1, SA 5, and SA 6) and methicillin-resistant *S. aureus* (SA 2, SA 3, and SA 4) strains and were identified by matrix-assisted laser desorption/ionization time-of-flight mass spectrometry as described in Rondevaldova et al. [90].

Mueller–Hinton (MH) broth was used as a cultivation medium, and both MH agar and MH broth purchased from Oxoid (Basingstoke, Hampshire, UK) were used as assay media. The pH of cation-adjusted MH broth was equilibrated to a final value of 7.6 with Trizma base (Sigma-Aldrich, Prague, Czech Republic). Stock cultures of bacterial strains were cultivated in broth medium at 37 °C for 24 h prior to the testing. The bacterial suspension’s turbidity used for the inoculation of both plate and lid, was adjusted to 0.5 McFarland standard by Densi-La-Meter II (Lachema, Brno, Czech Republic) to reach the final concentration of 10^7^ CFU/mL.

### 4.4. Antimicrobial Assay

The in vitro antibacterial combinatory potential of *O. vulgare* EO in combination with *T. vulgaris* EO in the liquid and vapor phase was determined using a broth volatilization checkerboard assay previously developed in our laboratory [17]. The method is based on the combination of classical microdilution checkerboard test and broth microdilution volatilization technique [16], allowing the determination of interactions between EOs and/or plant volatile agents simultaneously in liquid and vapor phase as well as comparison of MIC values and calculation of FICs in both liquid and solid media. Experiments were performed in white, 96-well immunoplates (total well volume = 400 µL) covered by tight-fitting lids with flanges designed to reduce evaporation (SPL Life Sciences, Naechon-Myeon, Korea). In the first part of the procedure, 30 µL of agar was pipetted into every flange on the lid (with the exception of the outermost wells) and inoculated with 5 µL of the bacterial suspension. Subsequently, both *O. vulgare* and *T. vulgaris* EOs were dissolved in DMSO and diluted in the broth medium to get the initial concentrations of 2048 µg/mL, with maximum DMSO content of 1%.

The preparation of plate assay and serial dilutions were performed by an automated pipetting platform, Freedom EVO 100, equipped with a four-channel liquid handling arm (Tecan, Mannedorf, Switzerland). In combinations, six two-fold serial dilutions of oregano EO from horizontal rows were subsequently cross-diluted vertically by six two-fold serial dilutions of thyme EO. The final volume in each well was 100 μL, except for the outermost wells, which were left empty to prevent edge leakage effect. The plates were subsequently inoculated by bacterial suspensions using a 96-pin multi-blot replicator (National Institute of Public Health, Prague, Czech Republic). Each plate also contained inoculated and non-inoculated broth, which served as growth and sterility controls, respectively. Oxacillin was used as a positive antibiotic control for verification of susceptibility of *S. aureus* strains in broth medium. The DMSO assayed as the negative control at a concentration of 1% did not inhibit any of *S. aureus* strains tested either in broth or agar media. After the inoculation, clamps (Lux Tool, Prague, Czech Republic) were used to fasten the plate and lid together, with handmade wooden pads (size 8.5 × 13 × 2 mm) for better fixing, and microtiter plates were incubated for 24 h at 37 °C.

MIC values and combinatory effects in both liquid and the vapor phases (i.e., in plates and on lids) were evaluated by visual assessment of bacterial growth after coloring of metabolically active staphylococcal colonies with 25 µL of MTT dye in a concentration of 600 µg/mL when the interface of color in broth and on agar changed from yellow and purple (relative to that of colors in control wells). The MIC values were defined as the lowest concentration that visually inhibited staphylococcal growth compared to the compound-free growth control and were expressed in µg/mL. The final MIC values presented in this work are the average of MICs obtained from three independent experiments that were performed in triplicate.

The combinatory effect of EOs was determined based on the value of ΣFIC. For the combination of agent A (*O. vulgare* EO) and agent B (*T. vulgaris* EO), the ΣFIC was calculated according to the following equation: ΣFIC = FIC_A_ + FIC_B_, where FIC_A_ = MIC_A (in combination with B)_ /MICA _(alone)_, and FIC_B_ = MIC_B (in combination with A)_ /MICB _(alone)_ and evaluated according to EUCAST [50]. The ΣFIC index was interpreted as follows: synergistic interaction if ΣFIC ≤ 0.5; additive effect if ΣFIC > 0.5 and ≤ 1; indifferent if ΣFIC > 1 and < 2; and antagonistic if ΣFIC ≥ 2.

### 4.5. GC/MS Analysis

For determination of the main components of *O. vulgare* and *T. vulgaris* EOs, GC/MS analysis was performed using the dual-column/dual-detector gas chromatograph Agilent GC-7890B system equipped with autosampler Agilent 7693, two columns (fused-silica HP-5MS column (30 m × 0.25 mm, film thickness 0.25 μm) and a DB-HeawyWAX column (30 m × 0.25 mm, film thickness 0.25 μm)) and a flame ionization detector (FID) coupled with a single quadrupole mass selective Agilent MSD-5977B detector (Agilent Technologies, Santa Clara, CA, USA). Operational parameters were helium as carrier gas at 1 mL/min, injector temperature 250 °C for both columns. The oven temperature was raised for both columns from 50 °C to 280 °C. Initially, after an isothermic period of 3 min, the heating rate was 3 °C/min until the temperature reached 120 °C. Subsequently, the heating velocity increased to 5 °C/min until it reached 250 °C; and after 5 min of holding time on 250 °C, the heating rate increased to 15 °C/min until it reached 280 °C. Heating was followed by an isothermic period of 20 min. Both EOs were diluted in *n*-hexane for GC/MS at a concentration of 20 µg/mL, and for quantitative analysis, 1 μL of methyl octanoate was added as an internal standard. One μL of each EO solution was injected in split mode (split ratio 1:50). The mass detector was set to the following conditions: ionization energy 70 eV, ion source temperature 230 °C, scan time 1 s, mass range 40–600 m/z.

Identification of constituents was based on a comparison of their retention indices (RI) and retention times (RT) and spectra with the National Institute of Standards and Technology Library ver. 2.0.f (NIST, USA) [91], as well as with authentic standards and literature [47,48,49,50,51,52,53,54,55,56]. The RI was calculated for compounds separated by both HP5-5MS and DB-HeawyWAX columns using the retention times of n-alkanes series ranging from C_8_ to C_40_ (Sigma-Aldrich, Prague, Czech Republic). For each analyzed EO, the final number of compounds was calculated as the sum of components simultaneously identified using both columns and the remaining constituents identified by individual columns only. Quantitative data were calculated according to Cachet et al. [70] using the following formula

(1) $$m_{i}{=\mathrm{RRF}}_{i}^{\mathrm{Pred}}m_{\mathrm{MO}}\frac{A_{i}}{A_{\mathrm{MO}}}$$

where $m_{i}$ is the mass of the compound i to be quantified, expressed in mg per 1 kg of plant dry weight (DWP); ${\mathrm{RRF}}_{i}^{\mathrm{Pred}}$—predicted relative response factor of compound i (calculated from the molecular formulae of the component using a methyl octanoate as internal standard), $m_{\mathrm{MO}}$—mass of methyl octanoate (internal standard, IS), Ai and A_MO_ are the peak areas of the analyte and the IS, respectively, determined by the FID. Moreover, relative percentage contents of identified components have been determined using the FID data and indicated for both columns.

## 5. Conclusions

In summary, the present study reports the results of antistaphylococcal interactions between EOs obtained from *O. vulgare* and *T. vulgaris* that were tested by broth volatilization checkerboard assay. This combination of volatile oils exhibited additive effects against all 12 *S. aureus* strains in both liquid and vapor phases, whereas the best results in the liquid phase were obtained against methicillin-resistant strain (SA 2). To the best of our knowledge, this is the first report on interactions between *O. vulgare* and *T. vulgaris* EOs against *S. aureus* in the gaseous phase. In addition, the results presented in the form of isobologram, a graphical diagram enabling precise and intuitive judgment of the additive effect produced by EOs combination, validates the accuracy of broth volatilization checkerboard method for evaluation of the combinatory antimicrobial effect of EOs in the vapor phase. These results can potentially serve as a base for further research focused on the development of various pharmaceutical applications that are based on volatile microbials. However, since the MICs values obtained in the gaseous phase are only indicative and the real concentrations of evaporated EOs are lower, we believe that our results suggest a potential of thyme and oregano combination for application in the inhalation therapy against respiratory infections caused by *S. aureus.* However, further research focusing on in vivo evaluation will have to be carried out in order to verify its potential practical use.

## Figures and Tables

**Table 1:** In vitro inhibitory activity of interactions between *Origanum vulgare* and *Thymus vulgaris* essential oils against *Staphylococcus aureus* in the vapor phase.

*S. aureus* Strains	MICs Alone (μg/mL)			OVEO at Concentration Indicated in MIC Column in Combination with Listed TVEO Concentrations (μg/mL)									
	OVEO	TVEO	O	+TVEO 512		+TVEO 256		+TVEO 128		+TVEO 64		+TVEO 32	
				MIC	ΣFIC	MIC	ΣFIC	MIC	ΣFIC	MIC	ΣFIC	MIC	ΣFIC
SA ATCC 25923	427	427	ND	16	1.24	59	0.74	149	0.65	242	0.72	299	0.78
SA ATCC 29213	683	569	ND	16	0.94	158	0.70	242	0.59	398	0.70	484	0.79
SA ATCC 33591	626	569	ND	16	0.94	112	0.63	270	0.67	313	0.61	370	0.65
SA ATCC 33592	796	484	ND	16	1.09	149	0.72	370	0.73	512	0.78	512	0.72
SA ATCC 43300	512	512	ND	16	1.03	92	0.68	228	0.69	398	0.90	455	0.95
SA ATCC BAA 976	484	484	ND	16	1.10	82	0.70	228	0.74	341	0.84	341	0.78
SA 1	683	512	ND	16	1.02	92	0.64	242	0.63	341	0.63	455	0.76
SA 2	683	626	ND	16	0.86	178	0.67	313	0.67	427	0.76	484	0.78
SA 3	455	455	ND	16	1.20	62	0.73	185	0.72	270	0.76	341	0.85
SA 4	484	484	ND	16	1.10	92	0.73	194	0.67	348	0.85	356	0.80
SA 5	427	427	ND	16	1.24	44	0.70	149	0.64	270	0.77	370	0.96
SA 6	740	796	ND	43	0.75	341	0.81	427	0.76	512	0.79	512	0.74

ATCC: American type culture collection; MIC: minimum inhibitory concentration, the values are expressed as an average from three independent experiments, each performed in triplicate (rounded to integers); ND: not determined; O: Oxacillin; OVEO: *O. vulgare* essential oil; SA: *Staphylococcus aureus*; TVEO: *Thymus vulgaris* essential oil; ΣFIC: sum of fractional inhibitory concentrations; the combinatory effect is evaluated as follows: synergy ΣFIC ≤ 0.5; additive ΣFIC > 0.5 and ≤ 1; indifferent ΣFIC > 1 and ≤ 2 (rounded to two decimal places).

**Table 2:** In vitro inhibitory activity of interactions between *Origanum vulgare* and *Thymus vulgaris* essential oils against *Staphylococcus aureus* in the liquid phase.

*S. aureus* Strains	MICs Alone (μg/mL)			OVEO at Concentration Indicated in MIC Column in Combination with Listed TVEO Concentrations (μg/mL)									
	OVEO	TVEO	O	+TVEO 512		+TVEO 256		+TVEO 128		+TVEO 64		+TVEO 32	
				MIC	ΣFIC	MIC	ΣFIC	MIC	ΣFIC	MIC	ΣFIC	MIC	ΣFIC
SA ATCC 25923	512	512	0.6	16	1.03	156	0.81	313	0.86	512	1.13	512	1.06
SA ATCC 29213	740	569	0.4	16	0.94	270	0.81	341	0.69	455	0.73	512	0.76
SA ATCC 33591	910	683	555	16	0.77	370	0.78	484	0.73	512	0.66	512	0.61
SA ATCC 33592	740	512	164	16	1.02	121	0.67	341	0.72	512	0.88	512	0.81
SA ATCC 43300	626	512	36	16	1.03	185	0.79	313	0.75	512	0.96	512	0.90
SA ATCC BAA 976	569	512	64	16	1.03	142	0.75	284	0.75	427	0.89	512	0.98
SA 1	512	512	6	16	1.03	128	0.75	284	0.81	512	1.13	512	1.06
SA 2	1024	967	149	16	0.55	427	0.68	512	0.63	512	0.57	512	0.53
SA 3	512	512	455	16	1.03	116	0.73	256	0.75	398	0.90	512	1.06
SA 4	512	512	427	16	1.03	121	0.74	256	0.75	484	1.07	512	1.06
SA 5	512	512	1	16	1.03	107	0.71	313	0.86	512	1.13	512	1.06
SA 6	967	796	1	44	0.75	356	0.72	512	0.71	512	0.62	512	0.58

ATCC: American type culture collection; MIC: minimum inhibitory concentration, the values are expressed as an average from three independent experiments, each performed in triplicate (rounded to integers with the exception of values lower than 1); O: Oxacillin; OVEO: *O. vulgare* essential oil; SA: *Staphylococcus aureus*; TVEO: *Thymus vulgaris* essential oil; ΣFIC: sum of fractional inhibitory concentrations; the combinatory effect is evaluated as follows: synergy ΣFIC ≤ 0.5; additive ΣFIC > 0.5 and ≤ 1; indifferent ΣFIC > 1 and ≤ 2 (rounded to 2 decimal places).

**Table 3:** Chemical composition of *Origanum vulgare* essential oil.

	^1^ RI		Component	^2^ C	^3^ RF	^4^ Column				^5^ Identification	
						HP-5MS		DB-H.WAX		HP-5MS	DB-H.WAX
	Obs.	Lit.				(%)	c	(%)	c		
1	922 ^a^	924	*α*-Thujene	Monoterpene hydrocarbon (MH)	0.765	1.17	0.19	0.77	0.13	GC/MS, RI	GC/MS
2	929 ^a^	932	*α*-Pinene	MH	0.765	0.67	0.11	0.42	0.07	GC/MS, RI, Std	GC/MS
3	945 ^a^	946	Camphene	MH	0.765	0.18	0.03	0.12	0.02	GC/MS, RI, Std	GC/MS
4	973 ^a^	974	*β*-Pinene	MH	0.765	0.16	0.03	0.10	0.02	GC/MS, RI, Std	GC/MS
5	991 ^a^	988	*β*-Myrcene	MH	0.765	1.87	0.31	1.23	0.21	GC/MS, RI	GC/MS
6	1005 ^a^	1002	*α*-Phellandrene	MH	0.765	0.14	0.02	0.08	0.01	GC/MS, RI	GC/MS
7	1009 ^a^	1008	3-Carene	MH	0.765	0.08	0.01	0.06	0.01	GC/MS, RI, Std	GC/MS
8	1017 ^a^	1014	*α*-Terpinene	MH	0.765	0.85	0.14	0.63	0.11	GC/MS, RI, Std	GC/MS
9	1028 ^a^	1025	*p-*Cymene	MH	0.698	8.25	1.24	5.63	0.88	GC/MS, RI, Std	GC/MS
10	1061 ^a^	1054	*γ*-Terpinene	MH	0.765	4.52	0.74	3.33	0.57	GC/MS, RI, Std	GC/MS
11	1078 ^a^	1068	*trans*-Sabinene hydrate	Oxygenated monoterpene (MO)	0.869	0.30	0.06	0.11	0.02	GC/MS, RI	GC/MS
12	1110 ^a^	1095	Linalool	MO	0.869	0.11	0.02	-	-	GC/MS, RI, Std	-
13	1185 ^a^	1165	Borneol	MO	0.869	0.06	0.01	0.58	0.11	GC/MS, RI, Std	GC/MS
14	1190 ^a^	1174	Terpinen-4-ol	MO	0.869	0.64	0.12	0.36	0.07	GC/MS, RI	GC/MS
15	1302 ^a^	1289	Thymol	MO	0.808	0.26	0.04	0.47	0.08	GC/MS, RI, Std	GC/MS
16	1314 ^a^	1298	Carvacrol	MO	0.808	77.92	13.52	82.60	15.01	GC/MS, RI, Std	GC/MS
17	1430 ^a^	1418	*β*-Caryophyllene	Sesquiterpene hydrocarbon (SH)	0.751	1.89	0.30	1.53	0.26	GC/MS, RI, Std	GC/MS
18	1466 ^a^	1452	Humulene	SH	0.751	0.26	0.04	0.18	0.03	GC/MS, RI	GC/MS
19	1517 ^a^	1505	*β*-Bisabolene	SH	0.751	0.45	0.07	0.35	0.06	GC/MS, RI	GC/MS
20	1181 ^b^	1185 ^c^	D-Limonene	MH	0.765	-	-	0.15	0.03	-	GC/MS, RI
21	1190 ^b^	1195 ^d^	*β*-Phellandrene	MH	0.765	-	-	0.15	0.03	-	GC/MS, RI
22	1438 ^b^	1445 ^e^	1-Octen-3-ol	Others (O)	0.748	-	-	0.22	0.04	-	GC/MS, RI
23	1450 ^b^	1450 ^f^	*cis*-Sabinene hydrate	MO	0.869	-	-	0.27	0.05	-	GC/MS, RI
24	1579 ^b^	1583 ^g^	Carvacrol methyl ether	O	0.798	-	-	0.36	0.06	-	GC/MS, RI
25	1848 ^b^	1868 ^h^	Carvacrol acetate	O	0.901	-	-	0.06	0.01	-	GC/MS, RI
26	1957 ^b^	1953 ^d^	Caryophyllene oxide	Oxygenated sesquiterpene	0.830	-	-	0.14	0.03	-	GC/MS, RI
Chemical classes											
Monoterpene hydrocarbons					17.89			12.67			
Oxygenated monoterpenes					79.29			84.39			
Sesquiterpene hydrocarbons					2.60			2.06			
Oxygenated sesquiterpenes					-			0.14			
Others					-			0.64			
**Total identified (%)**					**99.78**			**99.90**			

^1^ RI = retention indices; Obs. = retention indices determined relative to a homologous series of n-alkanes (C_8_–C_40_) on HP-5MS column and on DB-HeawyWAX column, Lit. = literature RI values [38], ^c^ [39], ^d^ [40], ^e^ [41], ^f^ [42], ^g^ [43], ^h^ [44]; ^2^ C = Class; ^3^ RF = Response factor (calculated from the molecular formulae of the component using a methyl octanoate as internal standard); ^4^ Column = composition of essential oil detected on HP-5MS and DB-HeawyWAX columns; (%) = relative percentage content; c = content is expressed as concentration in mg per 1 kg of dry plant material; - = not detected; ^5^ Identification method: GC/MS = Mass spectrum was identical to that of National Institute of Standards and Technology Library (ver. 2.0.f), RI = the retention index was matching literature database; Std = constituent identity confirmed by co-injection of authentic standards.

**Table 4:** Chemical composition of *Thymus vulgaris* essential oil.

	^1^ RI		Component	^2^ C	^3^ RF	^4^ Column					^5^ Identification
						HP-5MS		DB-H.WAX		HP-5MS	DB-H.WAX
	Obs.	Lit.				(%)	c	(%)	c		
1	922 ^a^	924	*α*-Thujene	Monoterpene hydrocarbon (MH)	0.765	0.93	0.14	0.55	0.11	GC/MS, RI	GC/MS
2	929 ^a^	932	*α*-Pinene	MH	0.765	1.01	0.15	0.67	0.13	GC/MS, RI, Std	GC/MS
3	944 ^a^	946	Camphene	MH	0.765	0.50	0.07	0.36	0.07	GC/MS, RI, Std	GC/MS
4	973 ^a^	974	*β*-Pinene	MH	0.765	0.24	0.04	0.18	0.04	GC/MS, RI, Std	GC/MS
5	991 ^a^	988	*β*-Myrcene	MH	0.765	2.71	0.40	1.35	0.26	GC/MS, RI	GC/MS
6	1005 ^a^	1002	*α*-Phellandrene	MH	0.765	0.16	0.02	0.12	0.02	GC/MS, RI	GC/MS
7	1008 ^a^	1008	3-Carene	MH	0.765	0.08	0.01	0.08	0.02	GC/MS, RI, Std	GC/MS
8	1017 ^a^	1014	*α*-Terpinene	MH	0.765	1.96	0.29	1.51	0.29	GC/MS, RI, Std	GC/MS
9	1029 ^a^	1025	*p*-Cymene	MH	0.698	24.08	3.22	18.00	3.22	GC/MS, RI, Std	GC/MS
10	1061 ^a^	1054	*γ*-Terpinene	MH	0.765	13.37	1.96	10.61	2.08	GC/MS, RI, Std	GC/MS
11	1078 ^a^	1068	*trans*-Sabinene hydrate	Oxygenated monoterpene (MO)	0.869	0.59	0.10	0.24	0.05	GC/MS, RI	GC/MS
12	1090 ^a^	1086	Isoterpinolene	MH	0.765	0.18	0.03	-	-	GC/MS, RI	-
13	1113 ^a^	1095	Linalool	MO	0.869	2.84	0.47	2.87	0.64	GC/MS, RI, Std	GC/MS
14	1149 ^a^	1141	Camphor	MO	0.887	0.28	0.05	0.30	0.07	GC/MS, RI, Std	GC/MS
15	1184 ^a^	1165	Borneol	MO	0.869	0.47	0.08	1.14	0.25	GC/MS, RI, Std	GC/MS
16	1190 ^a^	1174	Terpinen-4-ol	MO	0.869	0.91	0.15	-	-	GC/MS, RI	-
17	1244 ^a^	1232	Thymol methyl ether	Other (O)	0.798	0.77	0.12	2.06	0.42	GC/MS, RI	GC/MS
18	1254 ^a^	1244	Carvacrol methyl ether	O	0.798	0.64	0.10	1.47	0.30	GC/MS, RI	GC/MS
19	1289 ^a^	1285	Bornyl acetate	O	0.957	0.17	0.03	0.13	0.03	GC/MS, RI, Std	GC/MS
20	1302 ^a^	1289	Thymol	MO	0.808	42.34	6.55	48.46	10.04	GC/MS, RI, Std	GC/MS
21	1430 ^a^	1417	*β*-Caryophyllene	Sesquiterpene hydrocarbon (SH)	0.751	3.55	0.51	2.06	0.40	GC/MS, RI, Std	GC/MS
22	1466 ^a^	1452	Humulene	SH	0.751	0.10	0.02	0.10	0.02	GC/MS, RI	GC/MS
23	1478 ^a^	1475	Geranyl propionate	O	0.935	0.06	0.01	0.10	0.02	GC/MS, RI	GC/MS
24	1487 ^a^	1478	*γ*-Muurolene	SH	0.751	0.17	0.02	0.23	0.04	GC/MS, RI	GC/MS
25	1517 ^a^	1505	β-Bisabolene	SH	0.751	0.10	0.01	0.07	0.01	GC/MS, RI	GC/MS
26	1529 ^a^	1513	*γ*-Cadinene	SH	0.751	0.30	0.04	-	-	GC/MS, RI	-
27	1535 ^a^	1522	*δ*-Cadinene	SH	0.751	0.38	0.05	0.70	0.14	GC/MS, RI	GC/MS
28	1602 ^a^	1582	Caryophyllene oxide	Oxygenated sesquiterpene	0.830	0.37	0.06	0.40	0.09	GC/MS, RI, Std	GC/MS
29	1181 ^b^	1185 ^c^	D-Limonene	MH	0.765	-	-	0.31	0.06	-	GC/MS, RI
30	1192 ^b^	1199 ^c^	1,8-Cineole	MO	0.869	-	-	0.64	0.14	-	GC/MS, RI
31	1438 ^b^	1445 ^e^	1-Octen-3-ol	O	0.748	-	-	1.10	0.21	-	GC/MS, RI
32	1450 ^b^	1450 ^f^	*cis*-Sabinene hydrate	MO	0.869	-	-	0.68	0.15	-	GC/MS, RI
33	1471 ^b^	1475 ^d^	α-Copaene	SH	0.751	-	-	0.06	0.01	-	GC/MS, RI
34	1496 ^b^	1531 ^g^	Bourbonene	SH	0.751	-	-	0.07	0.01	-	GC/MS, RI
35	1799 ^b^	1804 ^h^	Calamenene	SH	0.707	-	-	0.07	0.01	-	GC/MS, RI
36	1824 ^b^	1840 ^i^	Thymol acetate	O	0.901	-	-	0.19	0.04	-	GC/MS, RI
37	2169 ^b^	2186 ^i^	Carvacrol	MO	0.808	-	-	2.65	0.55	-	GC/MS, RI
			**Chemical classes**								
			Monoterpene hydrocarbons			45.22		33.74			
			Oxygenated monoterpenes			47.43		56.98			
			Sesquiterpene hydrocarbons			4.60		3.36			
			Oxygenated sesquiterpenes			0.37		0.40			
			Others			1.64		5.05			
			**Total identified [%]**			**99.26**		**99.53**			

^1^ RI = retention indices; Obs. = retention indices determined relative to a homologous series of n-alkanes (C_8_–C_40_) on HP-5MS column and on DB-HeawyWAX column, Lit. = literature RI values [38] ^c^ [39], ^d^ [40], ^e^ [41], ^f^ [42], ^g^ [45], ^h^ [46], ^i^ [47] ^2^ C = Class; ^3^ RF = Response factor (calculated from the molecular formulae of the component using a methyl octanoate as internal standard); ^4^ Column = composition of essential oil detected on HP-5MS and DB-HeawyWAX columns; (%) = relative percentage content; c = content is expressed as concentration in mg per 1 kg of dry plant material; - = not detected; ^5^ Identification method: GC/MS = Mass spectrum was identical to that of National Institute of Standards and Technology Library (ver. 2.0.f), RI = the retention index was matching literature database; Std = constituent identity confirmed by co-injection of authentic standards.

## Data Availability

All data is contained within the article.
